# Improved Protocol for the Production of the Low-Expression Eukaryotic Membrane Protein Human Aquaporin 2 in *Pichia pastoris* for Solid-State NMR

**DOI:** 10.3390/biom10030434

**Published:** 2020-03-11

**Authors:** Rachel Munro, Jeffrey de Vlugt, Vladimir Ladizhansky, Leonid S. Brown

**Affiliations:** Departments of Physics, and Biophysics Interdepartmental Group, University of Guelph, 50 Stone Road East, Guelph, ON N1G 2W1, Canada; rmunro@uoguelph.ca (R.M.); jdevlugt@uoguelph.ca (J.d.V.); .

**Keywords:** biosynthetic isotope labeling, aquaporins, *Pichia pastoris*, solid-state NMR, membrane proteins, post-translational modification

## Abstract

Solid-state nuclear magnetic resonance (SSNMR) is a powerful biophysical technique for studies of membrane proteins; it requires the incorporation of isotopic labels into the sample. This is usually accomplished through over-expression of the protein of interest in a prokaryotic or eukaryotic host in minimal media, wherein all (or some) carbon and nitrogen sources are isotopically labeled. In order to obtain multi-dimensional NMR spectra with adequate signal-to-noise ratios suitable for in-depth analysis, one requires high yields of homogeneously structured protein. Some membrane proteins, such as human aquaporin 2 (hAQP2), exhibit poor expression, which can make producing a sample for SSNMR in an economic fashion extremely difficult, as growth in minimal media adds additional strain on expression hosts. We have developed an optimized growth protocol for eukaryotic membrane proteins in the methylotrophic yeast *Pichia pastoris*. Our new growth protocol uses the combination of sorbitol supplementation, higher cell density, and low temperature induction (LT-SEVIN), which increases the yield of full-length, isotopically labeled hAQP2 ten-fold. Combining mass spectrometry and SSNMR, we were able to determine the nature and the extent of post-translational modifications of the protein. The resultant protein can be functionally reconstituted into lipids and yields excellent resolution and spectral coverage when analyzed by two-dimensional SSNMR spectroscopy.

## 1. Introduction

Despite the significant recent progress in cryo-electron microscopy and X-ray crystallography, membrane proteins are still underrepresented in protein structural databases with only 991 unique membrane protein structures [1]. Even though membrane proteins represent more than 40% of drug targets [2,3], information on their structure and dynamics is often incomplete, especially when it comes to lipid-embedded proteins. Recent history of solid-state NMR (SSNMR) gives us many examples where this technique could dramatically advance the field. Membrane proteins reconstituted in lipids for SSNMR show increased protein stability as compared to solution NMR or X-ray crystallography; lipid bilayers also provide a native-like experimental environment [4,5,6,7,8,9,10]. In globular proteins, it has been found that magic-angle spinning SSNMR can resolve complete protein structures [11,12,13,14,15,16]. This has been adapted to membrane proteins, which are more challenging due to lower sensitivity, spectral degeneracy, and complicated sample preparation [4,6,7,17,18,19,20,21]. Structures solved using this technique include those of the microbial photosensor *Anabaena* sensory rhodopsin [22], chemokine receptor CXCR1 [23], Influenza A M2 channel [24], transmembrane protein CrgA from *Mycobacterium tuberculosis* [25], and outer membrane protein G [26]. These successes can be extended to other eukaryotic membrane proteins, some of which are biomedically relevant, but harder to express and reconstitute for SSNMR in a structurally homogeneous form [6,27,28,29,30,31,32,33].

For structural SSNMR studies, protein’s carbon and nitrogen atoms are isotopically labelled. In order to achieve this, the recombinant protein is expressed in minimal media [34,35,36,37]. This means that carbon and nitrogen are supplied by single controlled sources [35]. However, this can introduce stress into the host cells, reducing expression yields in comparison to enriched media production [38,39]. Therefore, it is critical to optimize the minimal media contents and growth conditions prior to switching to labelled minimal media. Once there is an established expression protocol, one can introduce various uniform and selective labelling schemes within the protein of interest by varying labeling of the carbon or nitrogen sources [11,40,41,42]. This can help with assigning chemical shifts, improve spectral resolution, and assist with collecting internuclear distance restraints [11,43].

The robust expression of milligram quantities of a structurally homogeneous protein and its functional reconstitution into a membrane-like environment are the key requirements for the successful structural and dynamic SSNMR analysis of a membrane protein [20,21,44,45]. Heterologous expression of eukaryotic membrane proteins can be especially challenging due to intrinsic low expression levels as well as native-folding and post-translational modifications that are often not supported by the *E. coli* expression system [39,46,47]. This is exemplified by the poor expression of G-protein coupled receptors (GPCRs) in *E. coli*-based systems [48,49,50,51,52,53,54,55]. As such, it is beneficial to use eukaryotic hosts that can suitably mimic the protein’s native expression conditions. Previously, the methylotrophic yeast *Pichia pastoris* has been used for producing eukaryotic membrane proteins for structural studies [56,57,58,59,60,61,62,63,64,65]. *P. pastoris* is an ideal host for the production of isotopically labelled proteins given its ability to perform post-translational modifications resulting in natively-folded eukaryotic proteins [66,67,68]. Additionally, there is a large number of established protocols for efficient and inexpensive isotopic labeling of proteins in *P. pastoris* and its usefulness as the expression host for numerous proteins for crystallographic structures [69,70,71].

The ability of *P. pastoris* to grow to high cell densities and its tightly regulated alcohol oxygenase promoter make it an attractive system for recombinant expression of eukaryotic proteins [72,73,74,75]. Additionally, transformed *P. pastoris* are very stable due to the homologous recombination of the gene of interest into the genome [66,76]. As such, one can screen for clones with multiple insertion events in which antibiotic resistance is proportional to the number of insertions, which can lead to greater recombinant protein yields [77,78,79]. Previously, we adapted protocols developed for isotopic labeling of soluble proteins in *Pichia* for the production of integral membrane proteins, namely fungal rhodopsin from *Leptosphaeria maculans* (LR) [31] and human aquaporin 1 (hAQP1) [30]. These protocols yielded high-resolution magic angle spinning (MAS) SSNMR spectra of uniformly, doubly (^15^N/^13^C) labeled protein, with expression yields of approximately 5 mg per liter of culture. However, when it comes to human membrane proteins, this success with hAQP1 may not be typical, and is partly due to its intrinsically high expression level and relative stability [80]. Other human aquaporins are more challenging to express for SSNMR studies, as they show reduced production relative to hAQP1 in fermenter-based *P. pastoris* cultures [81].

The human aquaporin family consists of 13 members designated 0 through 12, which have important roles in water transport and for some (hAQP3, hAQP7, hAQP9, and hAQP10), glycerol transport regulation throughout the human body [81,82,83,84]. They are homotetrameric proteins, and each monomer has its own water and/or glycerol channel. In this study, we expressed human aquaporin 2 (hAQP2), which is located in the apical plasma membrane of collecting duct cells and acts as a selective water channel responsible for 20% of water reabsorption in the nephron of the kidney [58,85]. hAQP2 concentration in the plasma membrane is tightly regulated through a vasopressin V2 receptor signal transduction pathway, and hAQP2 dysfunction has been linked to impaired cellular trafficking, which leads to nephrogenic diabetes insipidus (NDI) [86,87]. NDI manifests in three forms, namely X-linked, autosomal dominant, and autosomal recessive [88]. X-linked NDI is due to mutations in the arginine vasopressin 2 receptor, which initiates hAQP2 expression and trafficking to the plasma membrane when bound to arginine vasopressin [89,90,91,92]. Autosomal NDI is due to point mutations in hAQP2. In the dominant form of autosomal NDI, if one out of four monomers that comprise the tetramer is mutated, the entire unit is retained in the endoplasmic reticulum or Golgi body and subsequently degraded [90,93]. However, in 90% of autosomal NDI cases, this disease is recessive wherein all monomers are mutated and thus retained and degraded. Of the 52 mutants associated with autosomal recessive NDI (ARNDI), 44 retain water transport activity, which implies that these functional mutants can be rescued from the endoplasmic reticulum and trafficked to the plasma membrane by some therapeutic compound [89,94]. Potential future studies of these mutants by SSNMR are contingent upon obtaining sufficient quantities of hAQP2 both in the wild-type and mutant forms.

hAQP2 has been shown to express quite poorly as compared to hAQP1, even under fermenter conditions [81]. While both proteins are orthodox aquaporins, hAQP1 is naturally present in high quantities in the plasma membrane due to dense packing, complete trafficking to the plasma membrane, and low degradation [80]. This makes hAQP2 a challenging target for SSNMR sample development. Several structures for hAQP2 have previously been solved using X-ray crystallography; however, the long C-terminal tail was either truncated for stability or resulted in a long alpha-helix protruding into the cytoplasm (the truncated wild-type structure PDB ID 4NEF [58], and the full-length S256A mutant structure PDB ID 4OJ2 [95]). This tail is functionally important as the location of multiple phosphorylation, protein–protein interaction, and ubiquitination sites [96,97,98]. Notably, phosphorylation of S256 is necessary to induce the insertion of hAQP2 into the apical membrane in the nephron collecting duct, thus rendering it water permeable [99]. Other sites on the tail that can be phosphorylated include S261, S264, and T269; they have been linked to stabilizing ubiquitinated hAQP2, hAQP2 excretion in exosomes, and increasing retention time of hAQP2 in the apical plasma membrane, respectively [100]. As such, it is important to be able to study this protein in its full-length wild-type form. SSNMR is advantageous in this respect as the protein does not need to undergo crystallization, and thus the long C-terminus can be studied in a more native environment.

Expression of aquaporins in *Pichia* has been well studied and optimized in bioreactors to result in exceptional yields [77,80,81,101]. However, the use of fermenters may be cost-prohibitive in the case of uniform isotopic labeling because of the requirement of the continuous addition of isotopically labeled carbon sources during induction. Typical fermenter conditions for AQP’s in *P. pastoris* consume 60 g of glycerol and 200–400 mL of methanol for a 3 L culture [101]. In contrast, a previously published growth protocol in a 1 L shaker flask culture uses 6.35 g of glucose and 5 mL of methanol [30,32]. Methanol induces recombinant protein production in *Pichia* but is toxic to the cells in excessive amounts. The protein responsible for the first oxidation reaction in the methanol utilization pathway, alcohol oxidase I, has a low affinity for oxygen [102,103], and to process the methanol added to the culture to induce protein expression large amounts of oxygen are required to prevent cell death due to methanol toxicity. Fermenters are advantageous as oxygen addition can be controlled, which in turn allows for continuous addition of methanol and thereby increased expression.

Another strategy employed for fermenter cultures is sorbitol co-feeding wherein a mixed feed of the carbon sources sorbitol and methanol is given [101,104,105,106,107,108,109]. Sorbitol is an excellent additional carbon source as it does not repress gene expression and has been shown to increase biomass and decrease the presence of a protein degradation products [101,105]. The co-feeding procedure appears to reduce cellular stress and thereby reduces degradation and aggregation of recombinant proteins [109]. More specific to aquaporins, sorbitol has previously been seen to reduce degradation products during *P. pastoris* recombinant expression of hAQP10 [110]. In these studies, sorbitol was added in the feed mixture with methanol in a proportion of 60% sorbitol to 40% methanol [110]. However, this high concentration of sorbitol is cost-prohibitive for the production of isotopically labeled SSNMR samples, and one needs to test the optimum amount of sorbitol to add, as it is an additional expensive carbon source that must be supplied in its isotopically labelled form. As such, one must find a way to employ and optimize these strategies in minimal media shake flask cultures while reducing overall costs.

Previously, expression of a soluble extracellular domain of α-subunit of a *Mus musculus* muscle acetylcholine receptor in shake flask cultures was seen to increase in the presence of 0.5 g/L sorbitol [108]. In this case, the methanol to sorbitol ratio was 10:1 which suggests that sorbitol’s beneficial effect can be employed at a much smaller concentration.

Here, we report an optimized sample preparation procedure for full-length, wild-type hAQP2 expression for SSNMR in *Pichia pastoris*. This expression protocol referred to as low-temperature, sorbitol, equal volume induction (LT-SEVIN) combines low temperature post-induction growth, sorbitol supplementation, and higher cell density induction, which together can improve protein yield up to 10-fold. The protocol results in a structurally homogenous sample, which gives high resolution MAS-SSNMR spectra suitable for in-depth studies of structure and dynamics.

## 2. Materials and Methods

### 2.1. Materials

Common chemicals of reagent grade were purchased from either Fisher Scientific (Unionville, Ontario, Canada) or Sigma-Aldrich (Oakville, Ontario, Canada). The isotopically labeled compounds (^15^NH_4_)_2_SO_4_, ^13^C-sorbitol, ^13^C-methanol, and ^13^C6-glucose were obtained from Cambridge Isotope Laboratories (Andover, MA, USA). The Ni^2+^-NTA (nitrilotriacetic acid) agarose resin was purchased from Qiagen (Mississauga, Ontario, Canada). Lipids were purchased from Avanti Polar Lipids (Alabaster, AL, USA). Plasmid with hAQP2 sequence inserted was purchased from GenScript (Piscataway, NJ, USA).

### 2.2. Transformation

The gene encoding hAQP2 (UniProt ID: P41181) with a N-terminal 10xHis tag and a TEV cleavage site was codon-optimized for *P. pastoris* and ligated into the plasmid pPICZ B with no C-terminal tags. The plasmid was linearized with BstXI and transformed into the *P. pastoris* strain SMD1168H by electroporation. The electroporated cells were incubated at 30°C without shaking for 3 h and then plated on YPDS (1% (w/v) yeast extract, 2% peptone, 2% dextrose, 1 M D-sorbitol) plates with 0, 100, 200, and 500 µg/mL zeocin. Plates were incubated at 30°C for 5 days until transformant colonies appeared.

### 2.3. Colony Screening

Thirteen transformed colonies were re-plated on YPD (1% (w/v) yeast extract, 2% (w/v) peptone, 2% (w/v) dextrose) plates containing increasing amounts of zeocin (500, 1000, 1500, and 2000 µg/mL) to select for transformants with the highest copy number of the hAQP2 construct. The four colonies which grew on 2000 µg/mL zeocin were inoculated in 5 mL of BMD (0.8% (w/v) ammonium sulfate, 2.5% glucose, 0.34% (w/v) YNB without amino acids and ammonium sulfate, 4 × 10^–5^% (w/v) biotin, 100 mM potassium phosphate buffer, pH 6) and grown overnight at 30 °C at 300 rpm. Cultures were diluted to 25 mL and allowed to shake for an additional 24 h. Cultures were centrifuged at 1500× g for 10 min and the supernatant was discarded. The cell pellet was resuspended in 25 mL of BMM (0.8% (w/v) ammonium sulfate, 0.5% methanol, 0.34% (w/v) YNB without amino acids and ammonium sulfate, 4 × 10^–5^% (w/v) biotin, 100 mM potassium phosphate buffer, pH 6) and grown at 30 °C, 240 rpm for 24 h. Cell were collected by centrifugation and washed twice with MilliQ water.

Cell pellets were resuspended in 2 mL of breaking buffer (50 mM potassium phosphate, pH 7.5, 10% (w/v) glycerol, 2 mM EDTA, 1 mM PMSF) with 1 mL of acid-washed, ice-cold glass beads. The mixtures were vortexed with 1 min on/1 min off pulses for a total of 8 min “on”. The cells were centrifuged at 700× g and the supernatant collected. The supernatant was centrifuged at 13,000× g for 10 min and the new supernatant was discarded. The pellet was resuspended in 500 µL of 10% (w/v) SDS and incubated at 65 °C for 10 min. The mixture was again centrifuged at 13,000× g for 5 min and the supernatants were run on SDS-PAGE. The gel was transferred to a PVDF membrane by iBLOT (Invitrogen, Burlington, Ontario, Canada) and protein was detected by Western blot analysis (one-hour Western kit with TMB Substrate, GenScript) using Pierce 6x His epitope-tag IgG2b mouse antibody (ThermoFisher, Unionville, Ontario, Canada*)*. The Western blot was imaged using UVP ChemiDoc-It TS2, and the bands were analyzed using the software GelAnalyzer 19.1 (GelAnalyzer, Budapest, Hungary). The colony that resulted in the strongest band at ~25 kDa was selected for further growth and used for all subsequent experiments.

### 2.4. hAQP2 Expression Optimization

The hAQP2 producing colony was grown in the large scale according to the previously published protocol used for production of isotopically labeled protein in *Pichia* [30,32,42]. In brief, cells were grown to an OD_600_ of 8 in 250 mL of BMD. Cells were centrifuged at 1500× *g* for 10 min and resuspended in 1 L of BMM and incubated for 21 h at 30 °C at 250 rpm. Cells were broken, solubilized, and purified as described below. Yield was estimated at 280 nm using the extinction coefficient of 35,200 M^–1^cm^–1^, and purity was monitored by SDS-PAGE. Initial yields were 0.5 mg per 1 L culture.

In order to boost yields, induction conditions were modified and monitored by Western blot of isolated *Pichia* membranes after hAQP2 expression. Small-scale flask cultures were grown overnight in 25 mL of BMD at 30 °C at 300 rpm. Protein expression was induced at an OD_600_ of 2 (100 mL of BMM) and an OD_600_ of 8 (25 mL of BMM). Concentrations of 0.05 to 1 g/L of sorbitol in BMM in small-scale shake flask cultures, induced at OD_600_ = 8 were also tested. Sorbitol supplementation at 0.5 g/L was also tested for induction at OD_600_ = 2. Finally, induction temperature was reduced to 20.5 °C in combination with OD_600_ = 8 and 0.5 g/L sorbitol. Western blot analysis was done as previously described for colony screening. All cell pellets were broken in 2 mL of breaking buffer. Membranes were spun down at 13,000× *g*, and pellets were retained for analysis. Membranes were incubated with 200 µL of 10% (*w/v*) SDS for 10 min at 65 °C, centrifuged again to remove insoluble materials, and 20 µL was loaded on a gel. The gel was run at 110 V for 90 min and the Western blot developed as described above. Large scale cultures of both sorbitol, equal volume induction (SEVIN), and low-temperature (LT)-SEVIN growths were performed, and purified protein was analysed by both SDS-PAGE of solubilized protein and Fourier transform infrared (FTIR) spectroscopy of hAQP2 reconstituted into liposomes.

### 2.5. LT-SEVIN Large Scale Growth

The best hAQP2 producing colony found by Western blot analysis was used to inoculate 6 mL of BMD. The cells were grown at 30 °C, 300 rpm overnight. The overnight culture was divided into six 250 mL baffled flasks, and each culture was diluted to 50 mL and grown for another 24 h. The cultures were subsequently diluted to 250 mL of BMD and grown overnight until the OD_600_ reached 7. Cultures were centrifuged at 1500× *g* for 10 min and resuspended in six flasks with 250 mL of BMM with 0.05% (w/v) D-sorbitol. For isotopic labeling, ammonium sulfate, glucose, methanol, and sorbitol were added as ^15^N and ^13^C labeled compounds (Cambridge Isotope Laboratories, Tewksbury, Massachusetts, United States). The cultures were incubated at 30 °C, 300 rpm for 1 h. hAQP2 production was induced with the addition of methanol to a final concentration of 0.5% (v/v), and the cultures were incubated at 240 rpm for 24 h at the lowered temperature of 20.5 °C. Cells were collected by centrifugation at 1500× *g*, and the pellet was stored at −80 °C.

### 2.6. Cell Breakage

The cell pellet was initially resuspended in 20 mL of breaking buffer and 10 mL of acid-washed, ice-cold glass beads. The sample was vortexed with 1 min “on”/1 min “off” pulses for a total of eight minutes “on”. The sample was centrifuged at 700× *g* for 5 min and the supernatant was collected. For all subsequent breakage cycles, 10 mL of breaking buffer was added to the cell pellet, and the sample was vortexed with 1 min “on”/1 min “off” pulses for a total of four minutes “on”. The sample was again centrifuged at 700× *g* for 5 min and the supernatant collected. These two steps were repeated until the cells were entirely broken and no pellet remained after centrifugation.

The collected supernatants were centrifuged at 100,000× *g* for 45 min at 4 °C, and the membrane pellet was retained. The membranes were resuspended in 12.5 mL of membrane buffer (20 mM Tris-HCl, pH 8, 20 mM NaCl, 10% (w/v) glycerol, 2x Roche EDTA-free protease inhibitor) for every 3.5 g of membranes. The membrane slurry was kept at −80 °C prior to solubilization for storage.

### 2.7. Solubilization

The membranes were thawed to room temperature and diluted to 25 mL per 3.5 g of membranes (approximately 0.75 L of cell culture) with detergent stock buffer (20 mM Tris-HCl, pH 8, 300 mM NaCl, 50% (w/v) glycerol, 5% (w/v) n-dodecyl-β-D-maltoside (DDM)) to a final DDM concentration of 2.5% (w/v). The mixture was incubated for 2 h at 4 °C with slow stirring. Insoluble debris were spun down at 100,000× *g* for 30 min at 4 °C, and the supernatant was collected.

### 2.8. Purification

NTA-Ni^2+^-agarose resin (Qiagen) was batch bound to the protein for one-hour at room-temperature with gentle stirring. The resin was then washed in 10 column volumes (CV) of Buffer A (20 mM Tris-HCl, pH 8, 300 mM NaCl, 0.05% (w/v) DDM, 10% (w/v) glycerol, Roche EDTA-free protease inhibitor) with 50 mM imidazole. The protein was eluted with 30 mL of Buffer A with 300 mM imidazole. Protein concentration in the eluate was monitored by light absorption at 280 nm using the extinction coefficient of 35,200 M^–1^cm^–1^ (after subtraction of the buffer absorption). Purity of the protein was confirmed using SDS-PAGE. Mass spectrometry to confirm protein identity and locate post-translational modifications was performed at the University of Western Ontario. Matrix-assisted laser desorption/ionization time-of-flight (MALDI-TOF) spectrometry (Bruker Daltonics Reflex IV, Bruker, Billerica, Massachusetts, United States) was used on the purified protein. Electrospray ionization mass spectrometry (ESI-MS) (Thermo Scientific Orbitrap Elite, Unionville, Ontario, Canada) was used on purified hAQP2, which was run on SDS-PAGE and digested with either trypsin or chymotrypsin. ESI-MS on hAQP2 isolated from SDS-PAGE prior to and after peptide:N-glycosidase F (PNGase F) treatment was employed to determine whether the glycosylation present on the protein was N-linked or O-linked.

### 2.9. Functional Assay of hAQP2

In order to confirm functionality of the purified protein, stopped-flow hypertonic shrinking assays were used following the procedure used for hAQP1 with some modifications [30,80,111]. While heart polar lipids were used for SSNMR sample preparation (see below), they did not form liposomes when extruded. Instead, a commonly used eukaryotic membrane mimetic, a mixture of egg phosphatidylcholine (PC)/brain phosphatidylserine (PS) (Avanti Polar Lipids, Alabaster, Alabama, United States) was used, which previously worked well for hAQP1 in our group [30], whereas PC/cholesterol mixture was employed for hAQP2 previously [99]. PC/PS liposomes (9:1) (w/w) were prepared by reverse-phase evaporation [112] and extruded through polycarbonate Isopore filters (0.4 and 0.2 µm, Sigma-Aldrich, Oakville, Ontario, Canada) consecutively. The resulting liposome stock was mixed with purified hAQP2 in 0.05% (w/v) DDM at a lipid to protein ratio of 10:1 (w/w) and mixed overnight at 4 °C. In order to quickly remove detergent to prevent the formation of 2D-crystals, 0.8 g of Bio-beads SM2 (Bio-Rad, Mississauga, Ontario, Canada) were added for every 1 mL of the protein–lipid mixture and allowed to mix gently at 4 °C for 48 h. The proteoliposomes were extracted by syringe and centrifuged at 300,000× *g* for 1 h at 4 °C and resuspended in liposome buffer (20 mM Tris-HCl, pH 8.0, 150 mM NaCl) at 0.2 mg lipid/mL. Protein-free liposomes were mixed with detergent and then treated with Bio-beads to make a control sample. Average diameter of the liposomes (165 nm) and proteoliposomes (246 nm) was estimated by dynamic light scattering (Malvern Zetasizer, Malvern, United Kingdom). Water permeability experiments were performed using a stopped-flow spectrometer (SX20, Applied Photophysics, Leatherhead, United Kingdom). Water efflux from the vesicles was monitored by the increase in light-scattering at 480 nm upon hypertonic shock as a response to exposure to 180 mM sucrose at 20 °C. Mercuric inhibition of the hAQP2 proteoliposomes was used as an added control to test the functionality of the protein by incubating the proteoliposomes with 0.1 mM HgCl_2_ for 15 min prior to osmotic shock.

### 2.10. Lipid Reconstitution for SSNMR

The eluate was buffer exchanged to Buffer A without protease inhibitor and imidazole through centrifugation with Amicon Ultra (cut-off 10 kDa). The protein was concentrated to 2 mg/mL. A modified version of the 2D crystallization protocol used by Schenk et al. [113] was employed. Heart polar lipids (Avanti Polar Lipids, Alabaster, Alabama, United States) were added to the concentrated hAQP2 at a protein to lipid ratio of 2:1 (w/w). Additional DDM was added to the mixture to a final concentration of 0.8 mg/mL. The sample was incubated at 4 °C on an Orbitron rotator overnight. The protein and lipid mixture was put in a dialysis bag (12–14 kDa cut-off) and incubated at 4 °C with gentle stirring in 1 L of the dialysis buffer (20 mM MES, pH 6, 100 mM NaCl, 4 mM MgSO_4_, 4 mM histidine). The sample was left to dialyze for 10 days. Dialysis buffer was exchanged three times for a total volume of 4 L with the final 1 L of buffer omitting MgSO_4_ and histidine. The reconstituted protein–lipid complexes were collected by centrifugation at 100,000× *g* for 30 min at 4 °C. The sample was resuspended in MilliQ water in a water sonication bath and washed 3 times to remove trace salt. Then, 100 µg of the sample was dried onto a CaF_2_ window, and FTIR spectroscopy (Bruker Vertex80, Bruker, Billerica, Massachusetts, United States) was used to confirm yield and sample quality.

### 2.11. Sample Preparation for SSNMR

The pellet was washed 3 times with NMR buffer (20 mM Tris-HCl, pH 7, 10 mM NaCl, 1% (w/v) glycerol) and spun down at 150,000× *g* for 10 min at 4 °C. Then, 1 mL of NMR buffer was added to the pellet and then centrifuged at 900,000× *g* for 3 h. The buffer was exchanged, and the sample was centrifuged again for 3 h at 900,000× *g*, 4 °C. The excess buffer was removed, and the pellet was stored at −80 °C prior to packing. The sample was center packed in a thin-wall 3.2 mm SSNMR rotor.

### 2.12. SSNMR Spectroscopy

NMR experiments were performed on a Bruker Avance III spectrometer operating at a proton frequency of 800.230 MHz and equipped with a Bruker 3.2 mm EFREE magic angle spinning (MAS) ^1^H–^13^C–^15^N probe. The MAS frequency was 14.3 kHz, and the sample temperature was maintained at 5 °C in all experiments. Sample temperature was calibrated using neat methanol as an external reference [114]. A 1D cross polarization ^13^C spectrum was recorded using ^1^H/^13^C cross-polarization [115] optimized around the *n* = 1 Hartman–Hahn condition [116] with *ca*. 62.5 kHz radio-frequency (rf) power on ^13^C and with the rf field ramped linearly around 76.8 kHz on the proton channel. Protons were decoupled during ^13^C acquisition using 87 kHz SPINAL64 proton decoupling [117].

Then, 2D ^13^C–^13^C correlation spectra were recorded with 13 ms dipolar-assisted rotational resonance (DARR) mixing [118,119], with 2612 points in the direct (t_2_) and 2200 points in the indirect (t_1_) dimension, with t_1_ and t_2_ time increments of 6.5 and 8.4 μs. Twenty four scans per point were recorded with a recycle delay of 1.7 s. Carbon chemical shifts were indirectly referenced to 4,4-dimethyl-4-silapentane-1-sulfonic acid (DSS) by adjusting the chemical shift of ^13^C adamantane downfield peak to 40.48 ppm [120].

Additionally, 2D ^13^C–^13^C insensitive nuclei enhanced by polarization transfer (INEPT) [121] excitation combined with total through-bond correlation spectroscopy (TOBSY) [122] was used to record correlation spectra of mobile fragments of hAQP2. INEPT selectively excites mobile species whereas TOBSY establishes correlations between through-bond coupled carbon atoms. Similar acquisition parameters as in DARR experiment were used to collect 2D INEPT TOBSY correlation spectra.

## 3. Results

### 3.1. hAQP2 Expression Optimization

**Figure 1:**
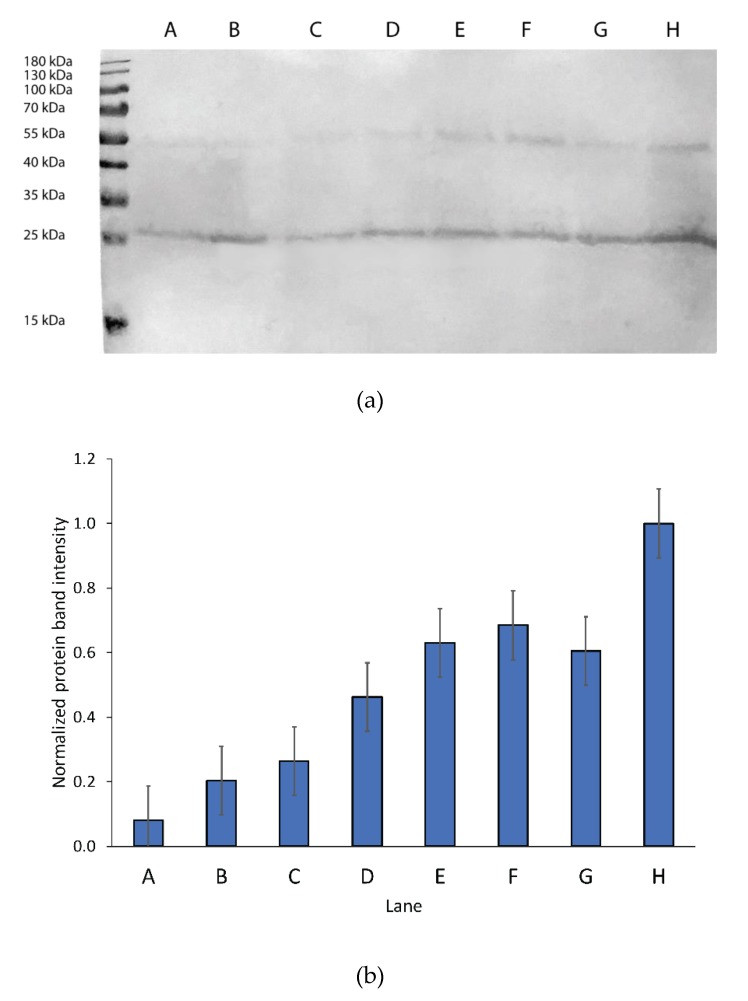
Comparison of hAQP2 expression under different growth conditions. (**a**) Western blot monitoring of hAQP2 expression under different induction conditions. The marker used was a Promega pre-stained protein ladder. All samples were grown in 25 mL of BMD media overnight at 30 **°**C prior to induction. lanes: A. Induction in 100 mL of BMM; B. Induction in 100 mL of BMM with 0.5 g/L sorbitol; C. Induction in 25 mL of BMM; D. Induction in 25 mL of BMM with 0.05 g/L sorbitol; E. Induction in 25 mL of BMM with 0.1 g/L sorbitol; F. Induction in 25 mL of BMM with 0.5 g/L sorbitol; G. Induction in 25 mL of BMM with 1 g/L sorbitol; H. Induction in 25 mL of BMM, at 20.5 **°**C with 0.5 g/L sorbitol. All cell pellets were broken in the same volume of breaking buffer. Membranes were centrifuged at 13,000 x *g* and the supernatant was removed. Membranes were incubated with 200 µL of 10% (w/v) SDS for 10 min at 65 °C, centrifuged again and 20 µL of the supernatant was loaded on a gel. The gel was transferred to a PVDF membrane by iBLOT (Invitrogen) and protein was detected by TMB substrate using the one-hour Western kit (GenScript) using mouse monoclonal 6xHis epitope-tag antibody (ThermoFisher). (**b**) Relative band intensity from the Western blot with LT-SEVIN (lane H) as the standard as estimated using GelAnalyzer for both the bands at 27 and 55 kDa. Band intensity was multiplied by 4 for lanes C through H to normalize for an induction volume of 100 mL of BMM to mimic the large-scale protocol.

In this optimized protocol, which allows economical isotope labeling of hard-to-express eukaryotic membrane proteins, we modified the induction step by introducing sorbitol co-feeding, higher cell density, and low-temperature induction. Western blot analysis of isolated membranes of hAQP2 producing cells under various expression conditions was used to monitor the additive effect of these modifications on hAQP2 yield per culture volume (Figure 1a). In previous protocols employed in our group for *Pichia* expression of membrane proteins in minimal media for solid-state NMR, we resuspended cells grown to an OD_600_ of 8 in 250 mL of BMD into 1 L of BMM (OD_600_ of 2) prior to induction [30,31,32]. We found a yield increase of 2-fold independent of sorbitol concentration when cells were resuspended in an equal volume of BMM (OD_600_ of 8) relative to BMD prior to induction. We also adopted the sorbitol addition approach used by Yao et al. for a soluble protein domain [108] to our membrane protein expression and observed that in the large-scale growth, the addition of 0.5 g/L sorbitol doubled the resultant biomass of hAQP2 producing cell-line measured after the cells were collected after induction. Western blot analysis performed after the addition of 0.5 g/L sorbitol in the classic growth protocol wherein cells grown in BMD were resuspended in a 4-fold volume of BMM for induction showed a similarly large yield increase (Figure 1b). Sorbitol concentration was optimized to reduce overall cost of the sample. To assess the effective sorbitol concentration range, we tested concentrations of 0.05 to 1 g/L of sorbitol in BMM in small-scale shake flask cultures, using equal volume induction conditions. Expression was seen to increase up to 8-fold relative to the traditional method with the addition of sorbitol up to 0.5 g/L; further increase in concentration of sorbitol showed no additional benefit (Figure 1). The purified protein yield was around 5 mg of hAQP2 per 1.5 L of minimal media LT-SEVIN growth culture as determined by a UV–Vis absorption peak at 280 nm with the extinction coefficient of 35,200 M^–1^cm^–1^. Yields were verified based on the amplitude of the amide I peak of the reconstituted protein as measured by FTIR. For comparison, the yield of codon-optimized hAQP2 in *P. pastoris* as grown according to the previously published protocols for SSNMR samples produced in *Pichia* [30,32,42] in 1 L of minimal media without sorbitol supplementation was 0.5 mg.

**Figure 2:**
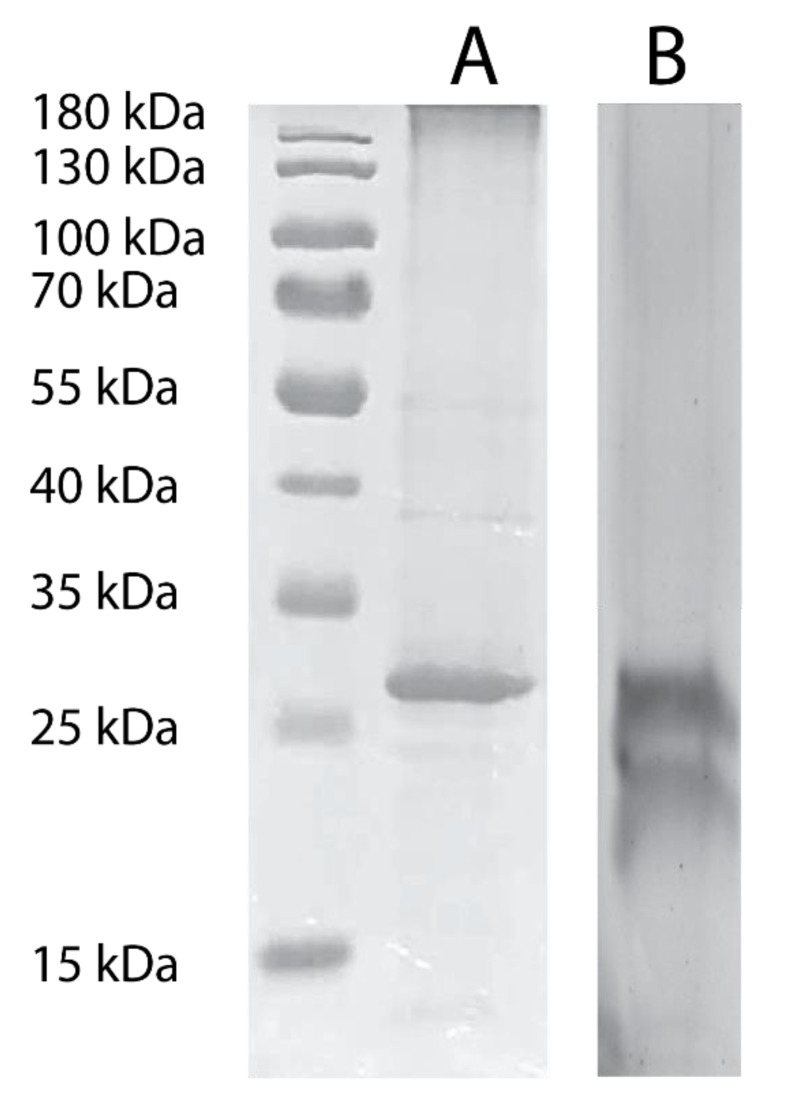
SDS-PAGE of purified hAQP2 as produced from 1.5 L cultures in (**A**) LT-SEVIN and (**B**) SEVIN growth protocols. The marker used was a Promega pre-stained protein ladder. Purified protein was concentrated to 2 mg/mL and 5 µL (**A**) or 10 µL (**B**) was incubated for 10 min at 65 °C with 5X SDS-PAGE loading dye (10% (w/v) SDS, 500 mM DTT, 50% (w/v) glycerol, 250 mM Tris-HCL, pH 6.8, and 0.5% (w/v) bromophenol blue dye). Samples were run at 110 V for 90 min, and bands were visualized with Coomassie Blue G-250 (Bio-Rad).

**Figure 3:**
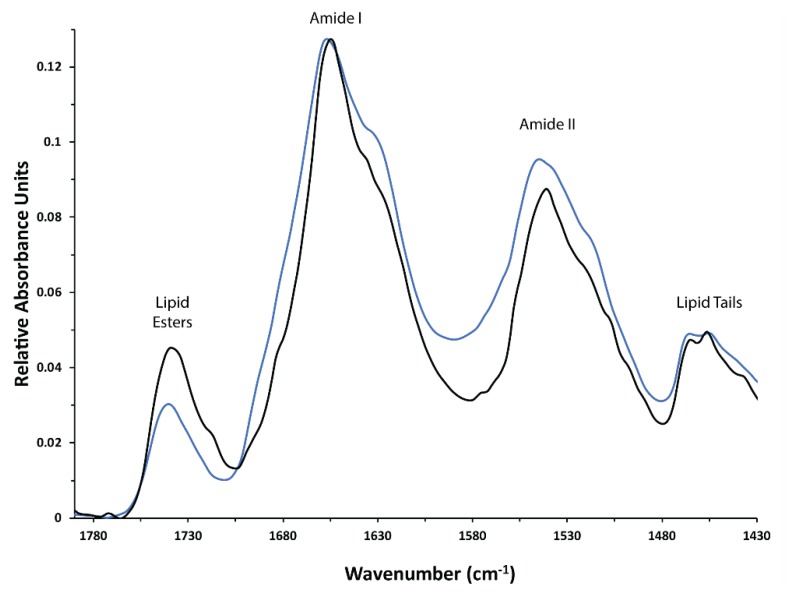
FTIR spectra of hAQP2 reconstituted into heart polar lipids at the target protein**/l**ipid ratio of 2:1 (w/w). hAQP2 expression in *P. pastoris* was induced at 30 **°**C (blue trace) and at 20.5 **°**C (black trace). Characteristic bands are labeled.

However, when grown in isotopically labelled media under sorbitol and equal-volume induction (SEVIN) conditions and purified according to the method published by Frick et al. [58] (see Materials and Methods above), both SSNMR and FTIR indicated significant structural heterogeneity in the sample reconstituted into heart polar lipids (see below). Furthermore, the SDS-PAGE analysis of the purified protein showed two bands, which were previously observed in hAQP2 samples and have been tentatively linked to differences in post-translational modifications or folding heterogeneity of the solubilized protein [123] (Figure 2). The reduction in induction temperature to 20.5 °C significantly reduced the heterogeneity in the sample, and resulted in largely alpha-helical (as judged from the amide I peak at 1655 cm^–1^ observed by FTIR [124,125]), structurally homogeneous sample (Figure 3). The reduction in the width of the amide I band in the low temperature (LT)-SEVIN sample as opposed to the SEVIN sample could be observed, along with reduction of the secondary amide I peak at ~1630 cm^–1^, which was associated with a beta-structure within the protein (Figure 3) [126]. SDS-PAGE of the LT-SEVIN produced purified protein showed a marked reduction in the lower molecular weight band while being of equivalent purity and yield (Figure 2). Additionally, a further yield increase of 50% (lane F as compared to lane H) could be estimated from Western blotting band intensity seen in the LT-SEVIN sample as compared to the SEVIN produced sample (Figure 1). Other modifications to the previous AQP purification and reconstitution protocols included using 2.5% (w/v) DDM as opposed to 2% (w/v) NG for solubilization, increasing glycerol concentration used throughout purification, and reconstitution in heart polar lipids instead of PC/PS used for the hAQP1 SSNMR sample [30,113]. Optimization of DDM and glycerol concentrations contributed to the stability of the purified protein and resulted in improvements in the FTIR spectra of reconstituted hAQP2 (not shown). DDM is a frequently used detergent for the isolation of membrane proteins, and the concentration was amended based on that used for G-protein coupled receptors [127]. Glycerol is a known protein-stabilizing compound that can help prevent aggregation and structural heterogeneity [128,129]. Most importantly, the decrease of induction temperature resulted in a dramatic increase of spectral resolution in SSNMR, yielding well-resolved spectra suitable for site-specific analysis (see below), in contrast to the poorly resolved spectra of the sample produced at 30 °C (not shown).

### 3.2. Functional Assays

**Figure 4:**
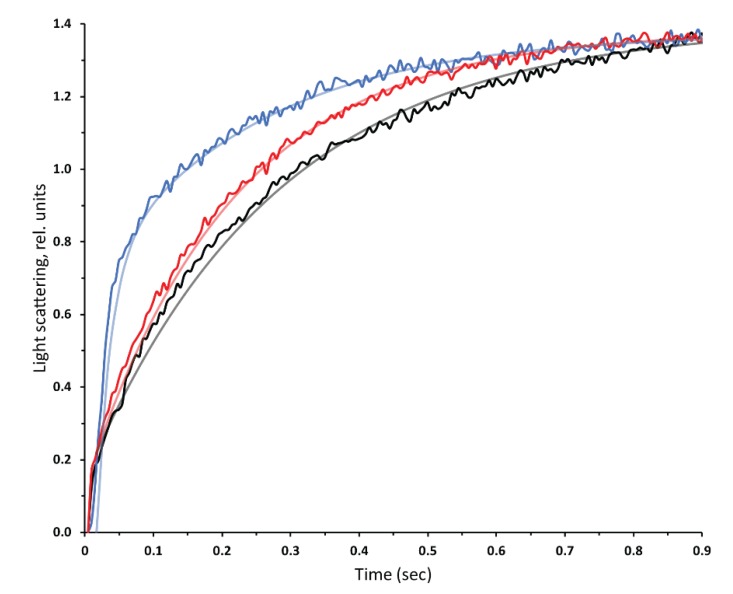
Kinetics of light scattering changes in PC/PS liposomes under hypertonic shock produced in the stopped-flow apparatus. Proteoliposomes reconstituted with hAQP2 at a protein:lipid ratio of 10:1 (w/w) (blue), the same proteoliposomes incubated with 0.1 mM HgCl_2_ (red), and PC/PS liposomes with no protein (black). Each trace is an average of 15 measurements and the exponential decay fits are displayed over the raw data (see text for details).

Stopped flow assays were used to confirm that *Pichia*-expressed hAQP2 was functionally active. PC/PS (9:1 (w/w)) lipids were prepared by reverse phase evaporation and extruded to form liposomes. Proteoliposomes were prepared at a high lipid to protein ratio (lipid/protein ratio of 10:1 (w/w)) as the presence of high protein concentrations, such as protein to lipid 2:1 (w/w) used for the SSNMR sample, results in leaky proteoliposomes [130]. Light-scattering changes upon osmotic shock were monitored in both the control liposomes and the proteoliposomes containing hAQP2 (Figure 4). The observed trends were very similar to those previously seen for hAQP1 [30]. Notably, proteoliposomes exhibited a much faster rate of shrinkage as compared to the control liposomes, suggesting the robust water transport through the protein (the difference in rates was even more marked when the shrinking rate was adjusted for the difference in the liposome and proteoliposome radii [111,131], which contributed a multiplication factor of ~1.5). For an additional control, the hAQP2 proteoliposomes were incubated in 0.1 mM HgCl_2_ for 15 min, and the experiment was repeated. This showed the expected marked reduction in the rate of water efflux, which is congruent with mercury being a known aquaporin inhibitor modifying a Cys181 within the aromatic/arginine constriction motif [132]. Similar to hAQP1, the shrinking kinetics of the control liposomes and the hAQP2 proteoliposomes inhibited by mercury could be approximated by single exponential decay functions, with characteristic time constants of 0.280 ± 0.005 s and 0.218 ± 0.002 s, respectively. The kinetics of shrinking of the proteoliposomes was markedly biphasic, displaying a major fast phase with 0.021 ± 0.001 s time constant (reflecting more than a ten-fold acceleration of water transport caused by hAQP2), and a minor phase with 0.244 ± 0.006 s time constant, which probably corresponded to either the protein-free fraction of liposomes or those reconstituted with non-functional fraction of hAQP2.

### 3.3. Analysis of Post-Translational Modifications by Mass Spectrometry

*Pichia pastoris* is known to produce varying patterns of proteolytic cleavage [133,134], glycosylation [135,136,137,138,139], and other post-translational modifications (PTMs) [140,141], which could affect the functional state of hAQP2 [97,99,100]. Mass spectrometry analysis was used to confirm protein identity and length, and to identify PTMs present in the protein. First, MALDI-TOF was used on the solubilized purified hAQP2, which was buffer exchanged to Buffer A without DDM and imidazole. This gave a molecular weight of 31,250 ± 30 Da, which corresponded to the expected weight of the expressed construct of 31,192 Da with the possible presence of some post-translational modifications, e.g., a phosphate that has a molecular weight of 80 Da, or an O-linked mannose (162 Da). Unfortunately, MALDI-TOF had insufficient resolution to give conclusive results on the exact nature of the PTMs, and we employed ESI-MS performed on the whole protein band excised from an SDS-PAGE gel. ESI-MS (in the positive ion mode) of the whole protein resulted in the four main peaks of 31,192 Da (major), 31,271 Da (major), 31,351 Da (minor), and 31,517 Da (minor), which corresponded to the expected full-length unmodified hAQP2 construct with all tags, the protein with one phosphate, the protein with two phosphates, and the protein with two sugar moieties of molecular 162.05 Da each, respectively. The mass of the sugar residues is what was expected for O-linked mannose, as the oxygen is donated from the Ser/Thr residue to which it is attached. The protein was also treated with PNGase F, which cleaves N-linked sugars, resulting in little changes to the ESI-MS spectra (not shown), strongly suggesting that the glycosylation was mainly O-linked. This is also consistent with the absence of noticeable GlcNAc additions (+203 Da), which would be expected for N-linked glycosylation [142].

**Figure 5:**
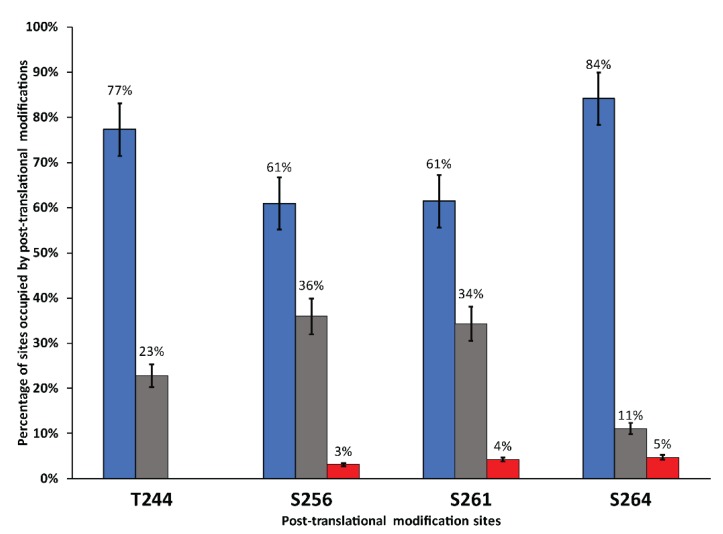
Percentage of peptides that contain post-translational modifications at T244, S256, S261, and S264 as predicted by ESI-MS of digested hAQP2. Unmodified sites are represented in blue, phosphorylated sites are black, and glycosylation sites are represented in red; 70 peptides covering the C-terminus were analyzed by ESI-MS.

These conclusions were confirmed using ESI-MS of trypsin and chymotrypsin digested hAQP2 isolated from the SDS-PAGE gel. PEAKS studio software (Bioinformatics Solutions Inc.) was used to analyze the mass of resultant peptides and determine possible locations of post-translational modifications based on the sequence of the hAQP2 construct. Phosphorylation was found in 60% of the analyzed peptides, which agreed with the peak corresponding to singly phosphorylated full-length protein in ESI-MS having the highest amplitude. Phosphates were found at four possible locations on the C-terminus of the protein (T244, S256, S261, and S264, which are known phosphorylation sites in humans [97,99,100,143]). O-linked mannoses were found to share S256, S261, and S264 with phosphates, with di-hexoses being predicted at S256 and S264. Based on the peptide molecular weights and the construct sequence, phosphorylation was predicted to be seen more often at S256 and S261 relative to T244 and S264 (Figure 5). Further PTM analysis, which confirmed the mass spectrometry results, was conducted by SSNMR (see below).

### 3.4. SSNMR Spectroscopic Characterization

**Figure 6:**
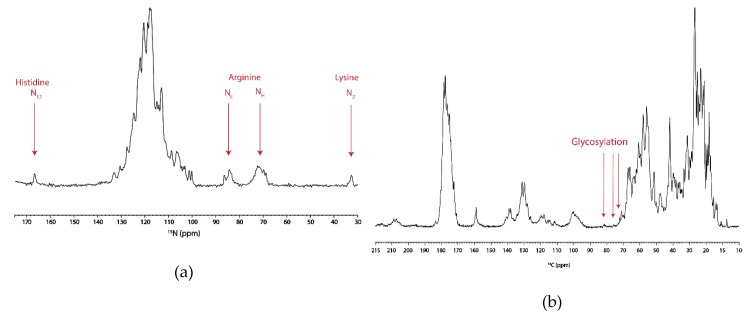
One-dimensional ^15^N (**a**), and ^13^C (**b**) MAS SSNMR spectra of ^13^C,^15^N-labeled hAQP2 reconstituted in heart polar lipids, recorded at 800 MHz, at 5 °C, and at a spinning rate of 14.3 kHz. The ^15^N spectrum was collected with 512 scans. The ^13^C spectrum was collected with 128 scans.

One-dimensional spectra for both ^15^N and ^13^C were collected to evaluate the quality of the hAQP2 SSNMR sample. The 1D-^15^N spectrum showed a good dispersion of backbone signals as well as resolved peaks for Lys, Arg, and His side-chain atoms (Figure 6a). The 1D-^13^C spectrum had similar resolution to the 1D-^15^N. However, the signals in the 70–80 ppm range are indicative of glycosylation, which is congruous with the mass spectrometry results (Figure 6b).

**Figure 7:**
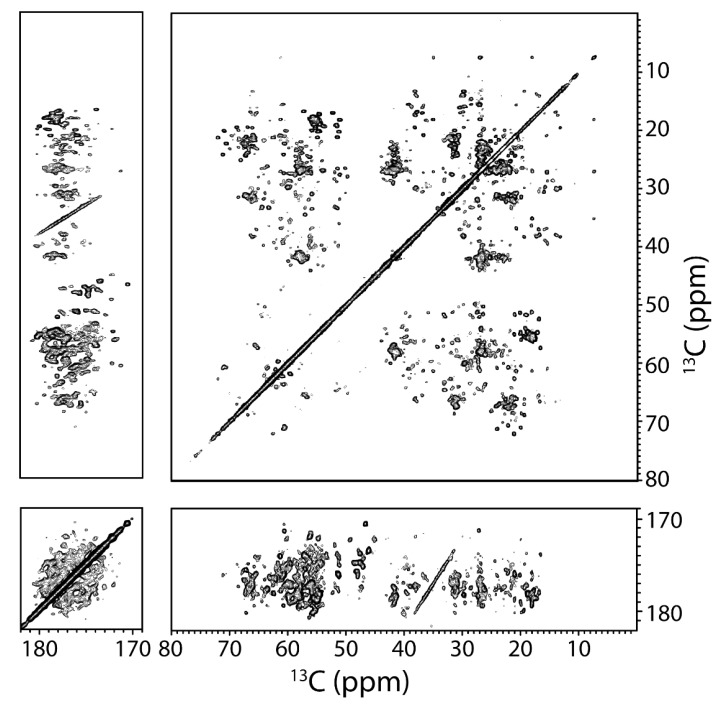
Two-dimensional ^13^C–^13^C DARR chemical shift correlation spectrum of ^13^C,^15^N-labeled hAQP2 proteolipid complexes at 800 MHz, at a spinning frequency of 14.3 kHz and at 5 °C. Mixing time was 13 ms. Data were processed with 40 Hz of Lorentzian line narrowing and 80 Hz of Gaussian line broadening in both dimensions. First contour level is cut at 5 × σ with each level multiplied by 1.15.

Next, a two-dimensional ^13^C–^13^C correlation DARR spectrum was collected on the UCN (uniformly carbon and nitrogen labeled) hAQP2 sample, which showed excellent spectral resolution with typical linewidths of 0.5 ppm indicative of structurally homogeneous sample (Figure 7). Characteristic amino acid intraresidue correlations could be used to identify cross-peaks belonging to several amino acid types such as Ala, Ser, and Thr. Additionally, a series of cross-peaks belonging to Ile located at 7.4, 17.9, 26.9, 34.9, and 61.2 ppm was similar to the Ile60 spin system seen in hAQP1 [144].

**Figure 8:**
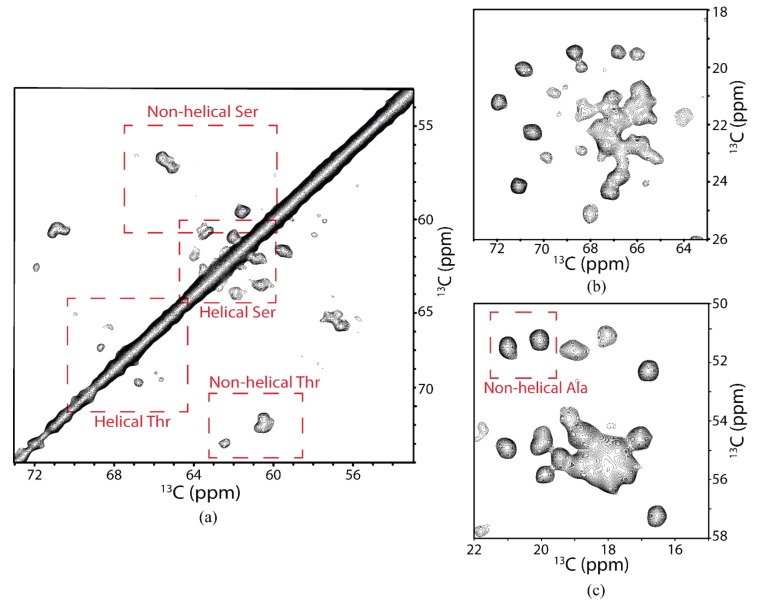
Selected characteristic regions of two-dimensional ^13^C–^13^C DARR chemical shift correlation spectrum of ^13^C,^15^N-labeled hAQP2 proteolipid complexes. Regions selected to show spectral coverage of (**a**) Ser and Thr CA/CB correlations, (**b**) Thr CA/CG and Thr CB/CG correlations, and (**c**) Ala CA/CB correlations.

**Figure 9:**
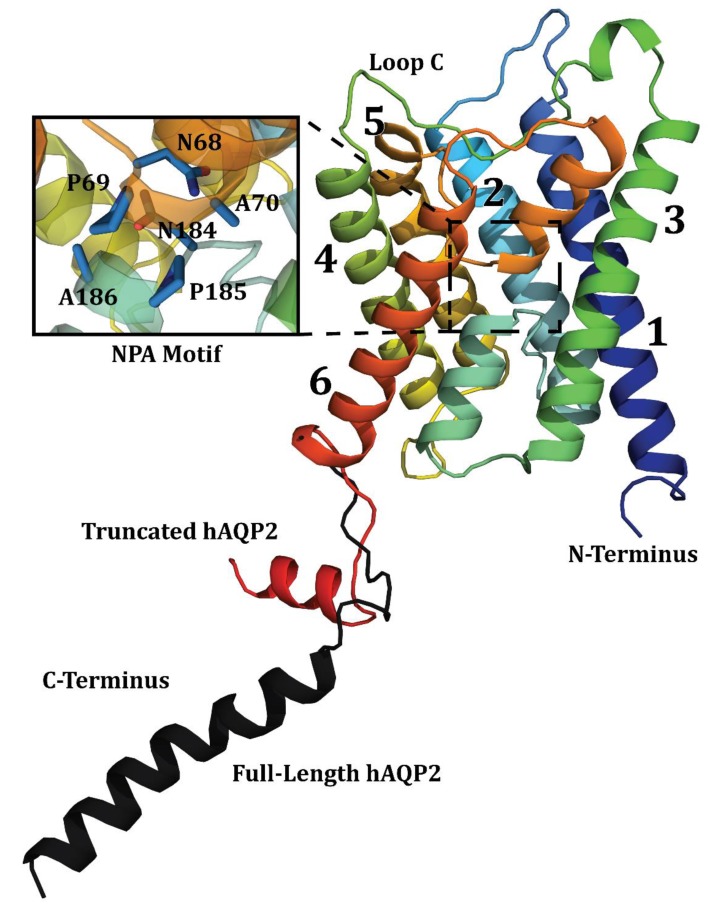
X-ray crystallographic structure of hAQP2 with major structural features identified. Helices are denoted 1–6 and loop C is indicated. Figure combines the truncated wild-type structure PDB ID 4NEF [58], and the C-terminal tail of the S256A mutant structure PDB ID 4OJ2 [95] (Black). Figure was generated in PyMOL (The PyMOL Molecular Graphics System, Version 2.0 Schrödinger, LLC ).

Spectral coverage for the identified systems corresponding to Ala, Thr, and Ser could be evaluated by estimating the integrated intensity of a region associated with specific amino acid cross-peaks and dividing by the intensity of well-resolved cross-peaks due to single residues. The CG–CA region for Thr showed an estimated total 10 correlations out of six TM domain residues and eight extramembrane residues as predicted by the X-ray crystallographic structure [58] (Figure 8b). Based on prior SSNMR experiments on microbial rhodopsins, it is less likely to see cross-peaks in 2D-DARR experiments for residues located in mobile regions of the protein due to unfavorable dynamics [145,146]. Therefore, some residues in the loop and tail regions of the protein must be sufficiently immobilized, e.g., by the secondary structure, to be visible in the 2D-DARR spectra. For example, loop C, which contained three Thr, is expected to be highly structured according to the X-ray crystallographic structure (Figure 9), as was also the case for hAQP1 [144,147]. Additionally, some loops, such as portions of loops B and E, re-enter the membrane to connect to the short half-helices forming the functionally important NPA (Asn-Pro-Ala) motifs located inside the transmembrane region, and were not likely to be mobile (Figure 9). This was confirmed in the region associated with Thr CA–CB correlations, which showed six well-resolved cross-peaks wherein two of the cross-peaks corresponded to chemical shifts associated with non-helical Thr (Figure 8a). As hAQP2 is a largely alpha-helical membrane protein, these non-helical Thr were likely to belong to loop regions that were sufficiently immobile. For Ala, we estimate that 16 helical CA–CB cross-peaks are present in the spectra, out of the total 35 in the protein (Figure 8c). Based on the available X-ray structure (PDB ID: 4NEF), only of them are expected to be in the TM regions, which confirmed excellent spectral coverage for the intramembrane Ala. There were two cross-peaks that corresponded to non-helical Ala residues, which could belong to the same structured loop regions as the observed non-helical Thr and non-helical Ser (Figure 8a,c).

**Figure 10:**
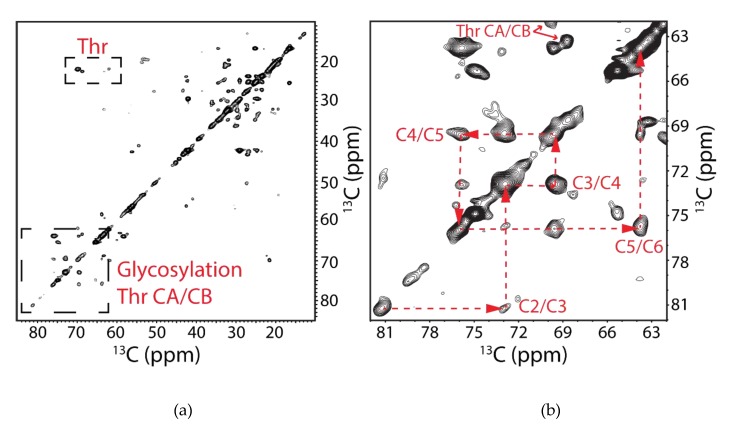
Two-dimensional ^13^C–^13^C INEPT TOBSY chemical shift correlation spectrum of ^13^C,^15^N-labeled hAQP2 proteolipid complexes at 800 MHz, at a spinning frequency of 14.3 kHz, and at 5 °C, showing the mobile parts of the protein and sugar moieties. (**a**) Spectrum between 10 and 85 ppm with the two regions of interest outlined in boxes. Signals corresponding to threonine CA/CG and CB/CG correlations as well the cross-peaks associated with glycosylation of hAQP2 are indicated. (**b**) Zoomed-in region between 62 and 82 ppm associated with the sugar cross-peaks with the spin system indicated with red dashed lines and labeled according to CASPER-determined α-D-mannose assignments. Data was processed with 40 Hz of Lorentzian line narrowing and 80 Hz of Gaussian line broadening in both dimensions. First contour level was cut at 5 × σ with each level multiplied by 1.1.

In order to identify the mobile regions of the protein, a 2D INEPT TOBSY spectrum was collected on the UCN hAQP2 sample. From this spectrum we could identify two strong cross-peaks which correlate to Thr CA/CG, as well as their CA/CB peaks (Figure 10a). Ten out of the fourteen Thr in the protein were observed in the DARR experiment; as such, we saw two of the expected four Thr residues. In addition to amino acid correlations, one could also identify several resonances that correlated with the presence of sugar moieties. Five resonances at 63.8, 69.5, 72.9, 75.9, and 81.3 ppm could be associated into a spin system of bonded carbon atoms (Figure 10b). Based on these chemical shift values, one could tentatively assign this sugar residue to α-D-mannose using the program CASPER [148,149], with C2 at 81.3 ppm, C3 at 72.9 ppm, C4 at 69.5 ppm, C5 at 75.9 ppm, and C6 at 63.8 ppm. However, this spectrum did not contain a cross-peak that corresponds to the anomeric carbon, typically located between 90 and 110 ppm. These shifts also match with previously assigned α-D-mannopyranoside disaccharides, which were found to be O-linked to yeast-expressed insulin-like growth factor I [150]. Our SSNMR results are in full agreement with the mass spectrometry data presented above, all pointing to the O-linked mannosylation of hAQP2.

## 4. Discussion

### 4.1. Post-Translational Modification of hAQP2

Both the SSNMR and mass spectrometry data suggest the presence of glycosylation in the hAQP2 sample. Despite the fact that glycosylation has not previously been observed in the *P. pastoris* expressed proteins LR [32] and hAQP1 [30] as produced for SSNMR by our group, it is not entirely unexpected. *P. pastoris* expressed recombinant proteins have been found to be glycosylated in many instances, usually by N-linked glycosylation [135,151,152]. In this glycosylation scheme, up to 40 mannose sugar moieties can be attached to a *Pichia*-expressed protein through a proximal N-acetylglucosamine (GlcNAC) [151,152,153]. Native glycosylation of aquaporins is also frequently observed, typically found on loop C, N-linked to an Asn residue [110,154,155]. This N-linked glycosylation has been commonly associated with proper trafficking of human aquaporins in vivo, as it acts as a signal that indicates that hAQP2 can exit the Golgi body and thus be trafficked [154]. As such, it was important to determine the nature and extent of this post-translational modification in our sample.

The chemical shifts we observed in the 2D INEPT TOBSY spectrum identify α-D-mannose as the likely glycosylating candidate; it is a sugar typically found in *Pichia*-expressed recombinant proteins. However, no chemical shifts were found that correspond to GlcNAc, precluding the presence of N-linked glycosylation. By combining the chemical shift data with the mass spectrometry results, it was determined that the LT-SEVIN-produced hAQP2 contains O-linked glycosylation. Given the molecular weight of the sugar adducts, di-mannose O-linked glycosylation is possible and has been observed previously on recombinant proteins expressed in yeast [137,150]. Furthermore, the chemical shifts of the sugar residue identified in 2D INEPT TOBSY corresponds very well to the primary mannopyranoside residue connected to a Thr (or Ser) of α-D-mannopyranoside disaccharides that were found O-linked to yeast-expressed insulin-like growth factor I [150]. Trypsin and chymotrypsin digestion and subsequent mass spectrometry analyses of the fragments suggest Ser residues on the C-tail of the protein as the likely sites of glycosylation, which agrees with the sugar cross-peaks being present in the INEPT TOBSY spectrum targeting mobile regions of the protein, unlike DARR, which does not show glycosylation in the immobile regions. However, these sites seem to be shared with phosphorylation sites also present on the tail, located at T244, S256, S261, and S264. ESI-MS of the protein showed a distribution of masses with the highest abundance belonging to singly phosphorylated hAQP2, which was primarily predicted to be located at S256 and S261. These sites are important for signaling and trafficking of hAQP2. S256 is the primary phosphorylation site associated with proper trafficking of hAQP2 to the apical membrane in vivo and is one of the sites associated with ARNDI mutations [97,99,100]. This site has also been hypothesized to affect water permeability of the protein [99]. Despite the random nature of the phosphorylation sites, the protein was functional when tested with a stopped-flow assay, and SSNMR spectral quality was not affected. In the future, mutants mimicking the unphosphorylated state and the phosphorylated state at these sites could be produced using LT-SEVIN to evaluate their possible effect on the structure and function of hAQP2. In the unpublished X-ray crystal structure of full-length hAQP2 (PDB ID 4OJ2) [95], S256 was mutated to an Ala residue, which resulted in a long helix extending into the cytoplasm. SSNMR of a similar sample could prove useful to determine whether this conformation is present in a more native lipid environment.

### 4.2. Expression of hAQP2

The LT-SEVIN protocol described above produced a sufficient quantity of homogenously structured uniformly isotopically labeled hAQP2 for multi-dimensional SSNMR experiments. HAQP2 typically expresses poorly even in enriched media and fermenter cultures [58,101]. The combination of sorbitol co-feeding, low temperature, and higher cell density at induction contribute greatly to the improved expression of stable protein under stressful growth conditions, in this case isotopic labeling on minimal media. By growing the *P. pastoris* culture to a higher density prior to induction in sorbitol-supplemented media, cell death is significantly reduced once methanol is added to stimulate hAQP2 expression. This can be attributed to the reduction in cellular stress during expression, which thereby reduces the amount of degradation to the expressed protein. The three protocol modifications are additive for boosting expression. By inducing at higher cell density, the liquid volume is significantly reduced, which allows for a higher oxygen to surface area ratio in the shaker flask cultures. This then reduces the toxicity of the methanol, which is added to stimulate hAQP2 production. The sorbitol addition further increases expression by providing a secondary carbon source, which reduces cell death and the presence of cell-degradation products. Sorbitol resulted in a cell biomass two times larger as compared to the traditional 1 L growth protocol used in the past for LR [32] and hAQP1 [30]. The combination of equal volume induction and sorbitol supplementation resulted in an over eight-fold increase in hAQP2 expression for a 1 L culture. However, the resultant protein was structurally heterogeneous and likely mis-folded. This was addressed by lowering the induction temperature to 20.5 °C, which gave a further yield increase of 50% relative to the SEVIN protocol alone. The resultant protein was structurally homogeneous as monitored by both FTIR and SSNMR of the reconstituted proteoliposomes. Lower induction temperatures have been shown to be useful in the case of complex proteins that misfold in recombinant conditions at high temperatures [156,157,158]. Some protocols use temperatures as low as 20 °C. The improved yields at 20.5 °C can possibly be attributed to lowering the speed at which the molecular machinery produces the protein, which is especially important in the case of membrane proteins as membrane insertion is directly linked to protein translation. This thereby lowers the likelihood of translational errors or misfolding occurring.

SSNMR of hAQP2 showed excellent spectral resolution in the collected 2D DARR correlation spectrum. Additionally, the spectrum shows good coverage of residues such as Thr, Ala, and Ser. These properties make this sample, as prepared by the LT-SEVIN expression protocol, a good candidate for further SSNMR analysis. The INEPT TOBSY experiment showed the presence of glycosylation, which was detected through mass-spectrometry analysis to be located on the C-tail region of the protein. This is significant, as the C-tail of hAQP2 is biomedically important, being involved in many protein–protein interactions and is the location of other post-translational modifications such as phosphorylation [97,99,100,155]. Previous X-ray structures of hAQP2 either excluded the tail or indicated it was a long helix protruding into the cytoplasm (PDB ID 4NEF [58] and PDB ID 4OJ2 [95]). Using INEPT TOBSY, we were able to observe post-translational modifications and residues located in this region under native-like conditions. Future experiments, such as production of isotopically labelled ARNDI-associated mutants for SSNMR, for example S226A or S256D, are contingent upon having the full-length protein. ESI-MS confirms the full-length protein is present and none of the C-tail residues has been truncated, which would exclude LT-SEVIN produced hAQP2 from future analysis of protein–protein interactions.

## 5. Conclusions

In this paper we presented a modified growth protocol for the expression and economical isotope labeling of recombinant membrane proteins in the yeast *P. pastoris*. This protocol combines higher cell density, sorbitol supplementation, and low temperature conditions at induction, resulting in a 10-fold increase in protein yield in shaker flask cultures. This provides an economically feasible methodology to produce isotopically labeled membrane protein samples for NMR. Furthermore, the produced protein (hAQP2) proved to be full-length, functional, stable, and structurally homogeneous when reconstituted into liposomes, as shown through SSNMR, stopped-flow assays, and FTIR. This protocol did result in post-translational modifications to hAQP2, notably phosphorylation and glycosylation. These post-translational modifications were all located in the C-terminal tail region of the protein, and shared four main sites, namely T244, S256, S261, and S264. These sites are biomedically important for hAQP2 function and have been shown to be modified in vivo [94]. The ability to monitor these sites is critical for future analysis of hAQP2 as the tail structure has not yet been satisfactorily solved. However, none of the modifications seem to affect the overall structural homogeneity of the protein sample, as spectral coverage and resolution proved to be excellent in two-dimensional SSNMR, both in DARR and INEPT-TOBSY. Overall, *Pichia*-expressed hAQP2 is an excellent candidate for future SSNMR studies, and LT-SEVIN is promising as a new protocol that can help express challenging proteins for SSNMR.
